# Supplementary material for: Phosphoproteomic screening identifies physiological substrates of the CDKL5 kinase
Source: EMBO J. 2018 Sep 28;37(24):e99559. doi: 10.15252/embj.201899559 (PMC6293279; doi:10.15252/embj.201899559)
Supplement: Supplementary file 1 — Appendix [file EMBJ-37-e99559-s001.pdf]

Appendix Table of Contents

Appendix Figures and Legends

Appendix Figure S1 Disruption of *CDKL5* in human U2OS Flp-In™ T-REx™ cells

Appendix Figure S2. Histograms and multi-scatter plots of phosphoproteomics data

Appendix Figure S3. Phosphomotif enrichment.

Appendix Figure S4. Data quality control of TMT labelling from FLAG-tagged MAP1S, CEP131 and DLG5 immunoprecipitates for XIC analysis

Appendix Figure S1

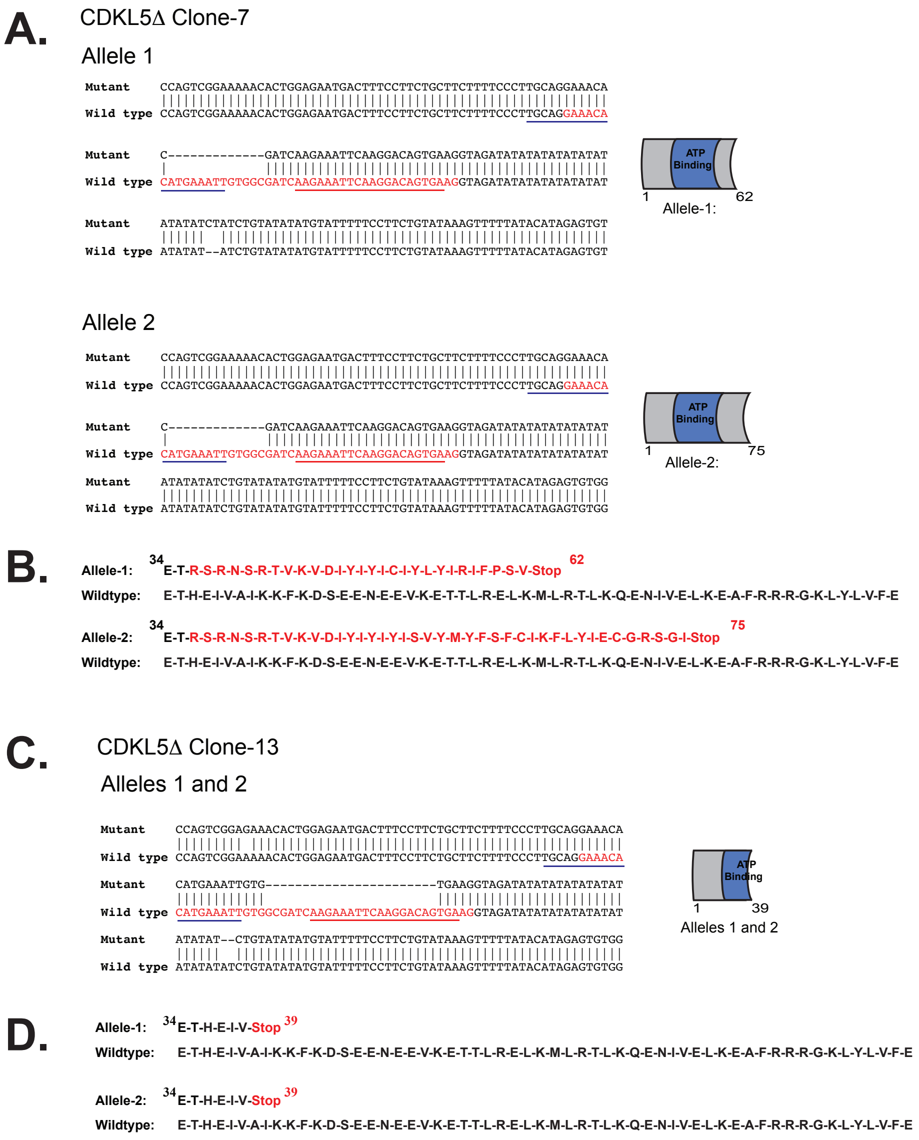

Appendix Figure S1. Disruption of *CDKL5* in human U2OS Flp-In™ T-REx™ cells

A. The sequence of the two *CDKL5* alleles in U2OS Flp-In™ T-REx™ cells *CDKL5* knockout clone 7 is aligned with the sequence of *CDKL5* from control cells. Exon 5 is highlighted in red lettering. The binding site of the one guide RNA is underlined with a red line, and the binding site of the other guide RNA is underlined with a blue line. Schematic diagrams on the right-hand side show the truncating effect on the CDKL5 protein product expressed from each allele.

B. The predicted protein sequence in the region of CDKL5 affected by the two different mutations in the two *CDKL5* alleles in knockout clone 7.

C. The sequence of the two *CDKL5* alleles in U2OS Flp-In™ T-REx™ cells *CDKL5* knockout clone 13 is aligned with the sequence of *CDKL5* from control cells. Exon 5 is highlighted in red lettering. The binding site of the one guide RNA is underlined with a red line, and the binding site of the other guide RNA is underlined with a blue line. Schematic diagrams on the right-hand side show the truncating effect on the CDKL5 protein product expressed from each allele.

D. The predicted protein sequence in the region of *CDKL5* affected by the mutation in both alleles in knockout clone 13.

Appendix Figure S2

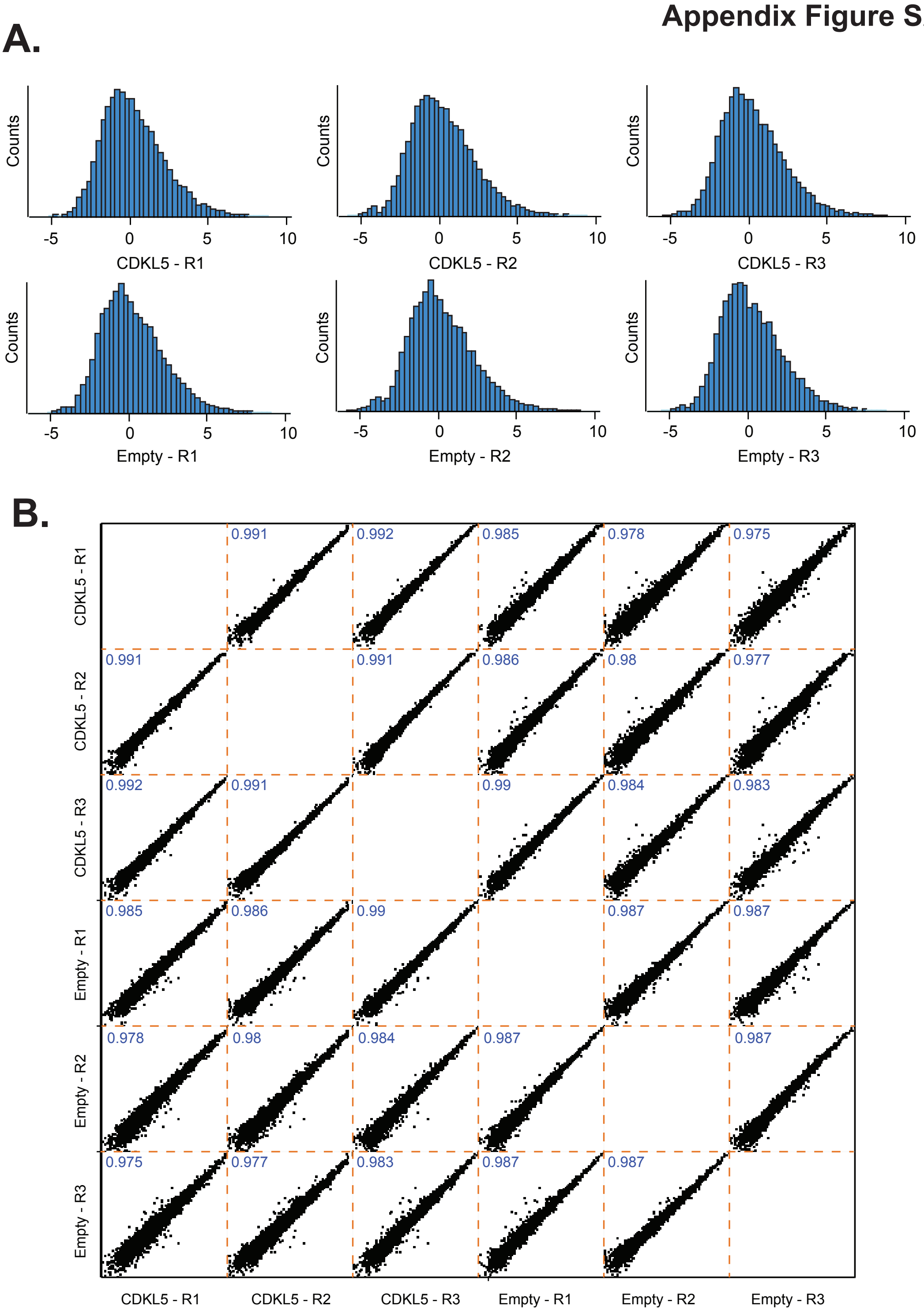

Appendix Figure S2. Histograms and multi-scatter plots of phosphoproteomics data

A. Histograms of the non-normalised phosphoproteomics data show high levels of reproducibility and normal distribution of data.

B. Multi-scatter plots of the comparisons between CDKL5-expressing and knock-out (empty) cells shows high levels of reproducibility. “R”: replicate.

Appendix Figure S3

A. Phosphothreonine

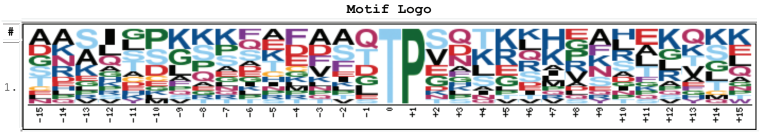

#	Motif	Fold Increase	Motif Score	Foreground Matches	Foreground Size
1.	...............TP..............	7.58	10.08	15	28

B. Phosphoserine

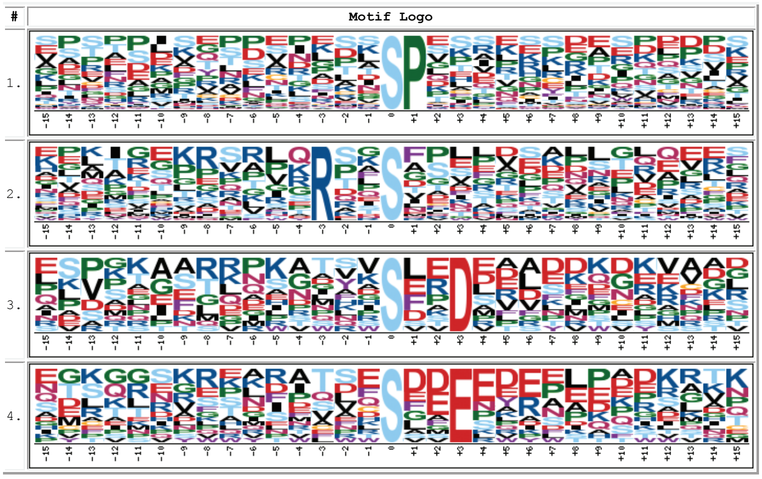

#	Motif	Fold Increase	Motif Score	Foreground Matches	Foreground Size
1.	...............SP..............	3.87	15.48	47	159
2.	............R..S...............	5.11	14.95	34	112
3.	...............S..D............	3.84	4.85	14	78
4.	...............S..E............	3.51	5.11	16	64

Appendix Figure S3. Phosphomotif enrichment.

Phosphopeptides that are more abundant in CDKL5 knockout cells expressing cells compared to empty vector control. (A) Phosphothreonine-containing motif. (B) Phosphoserine-containing motifs.

A.

Appendix Figure S4

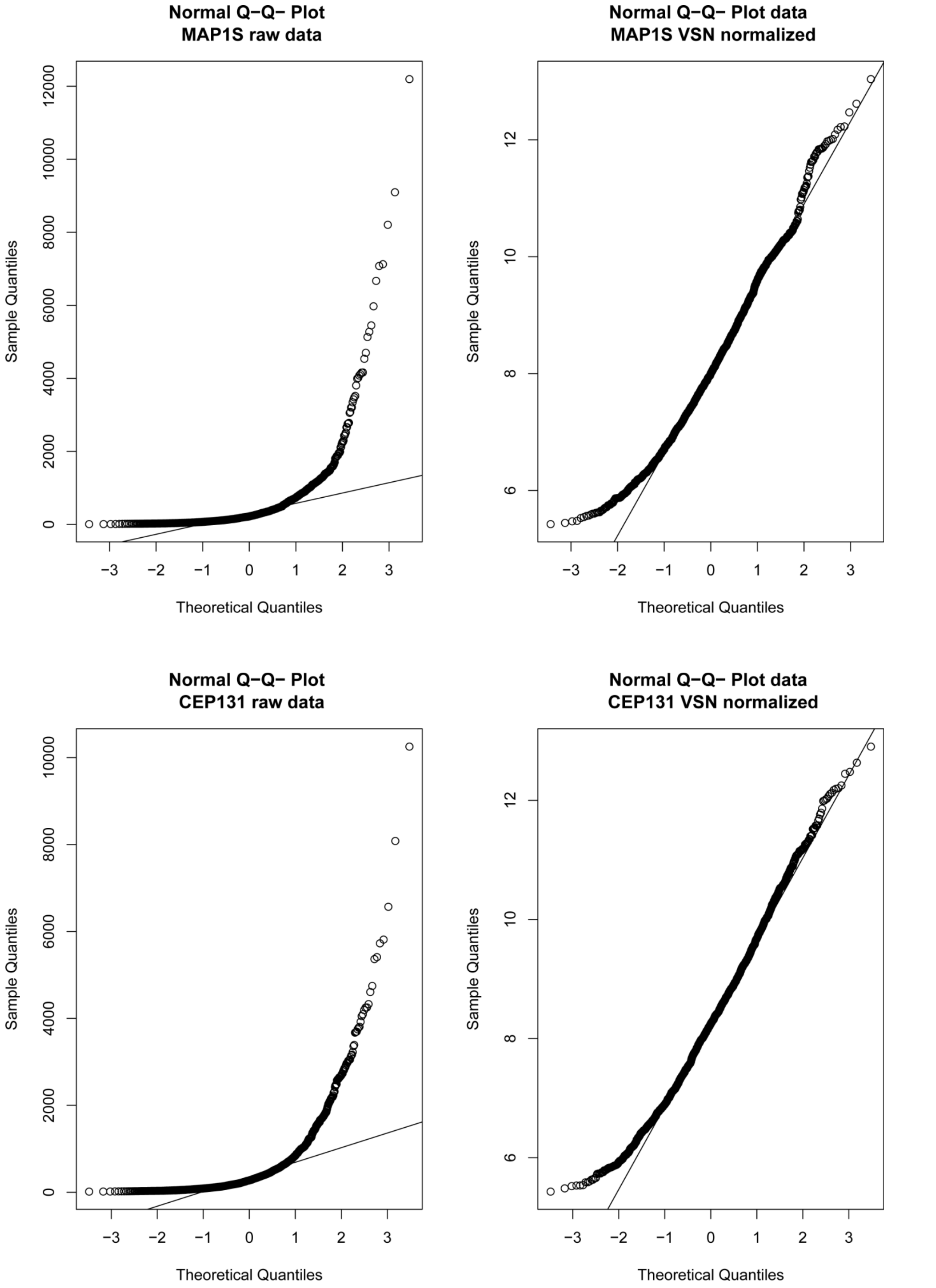

Appendix Figure S4

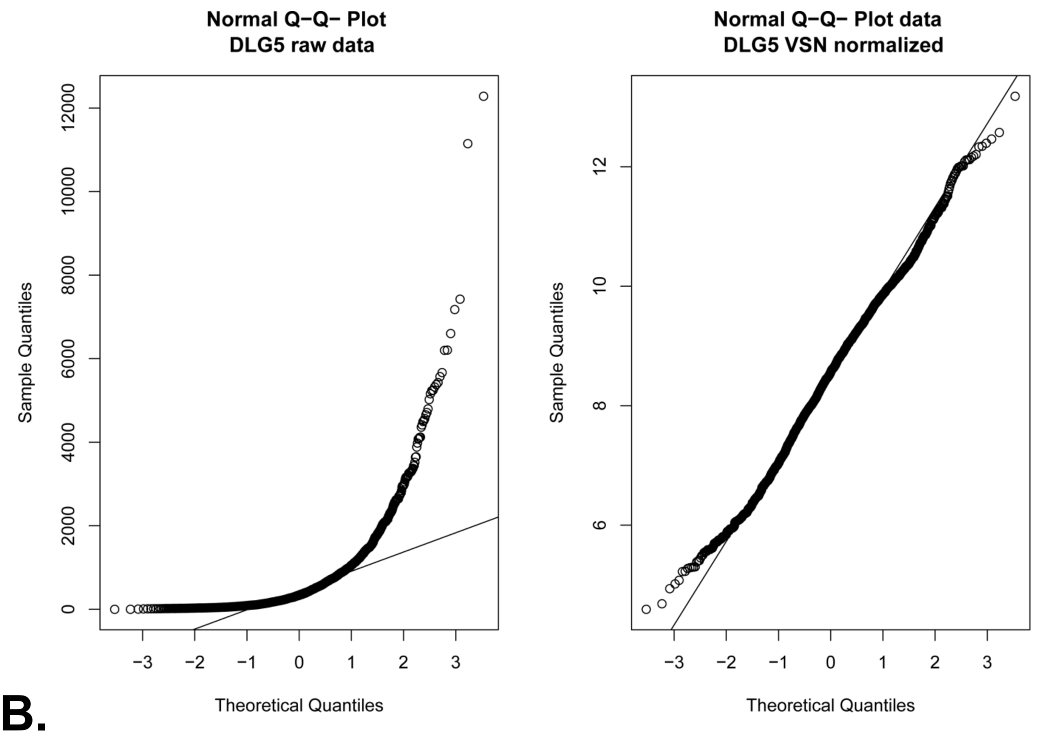

B.

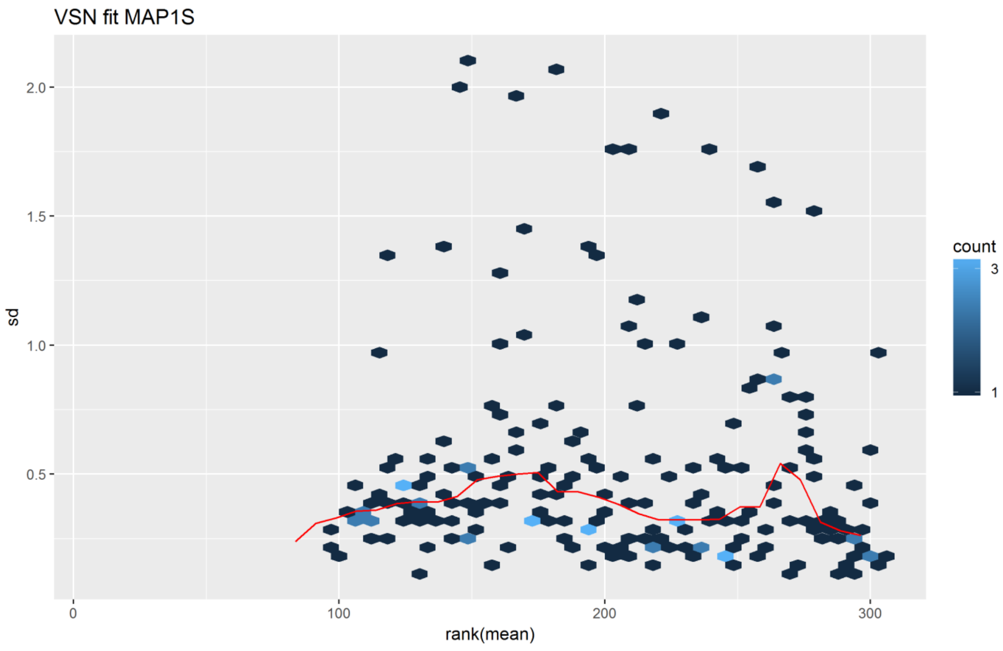

Appendix Figure S4

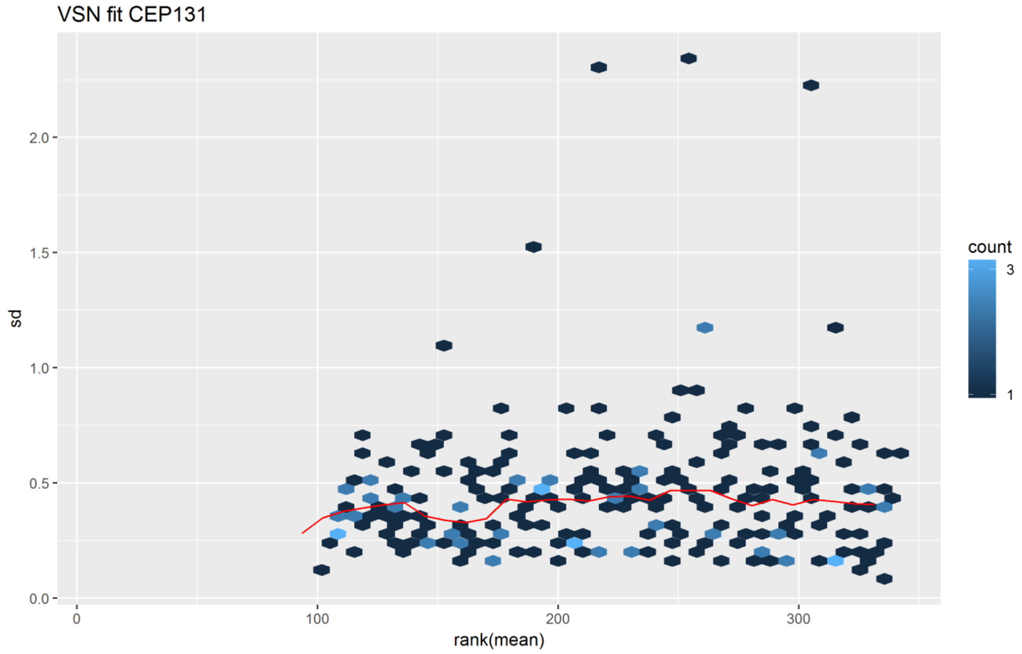

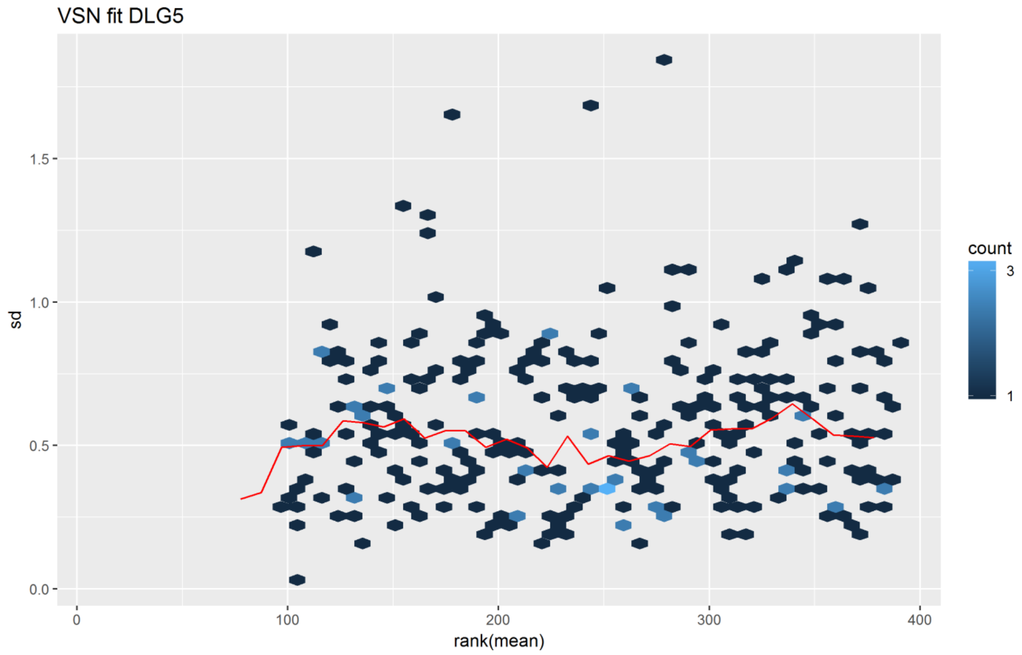

Appendix Figure S4

C.

MAP1S - VSN normalized intensities over all used TMT channels

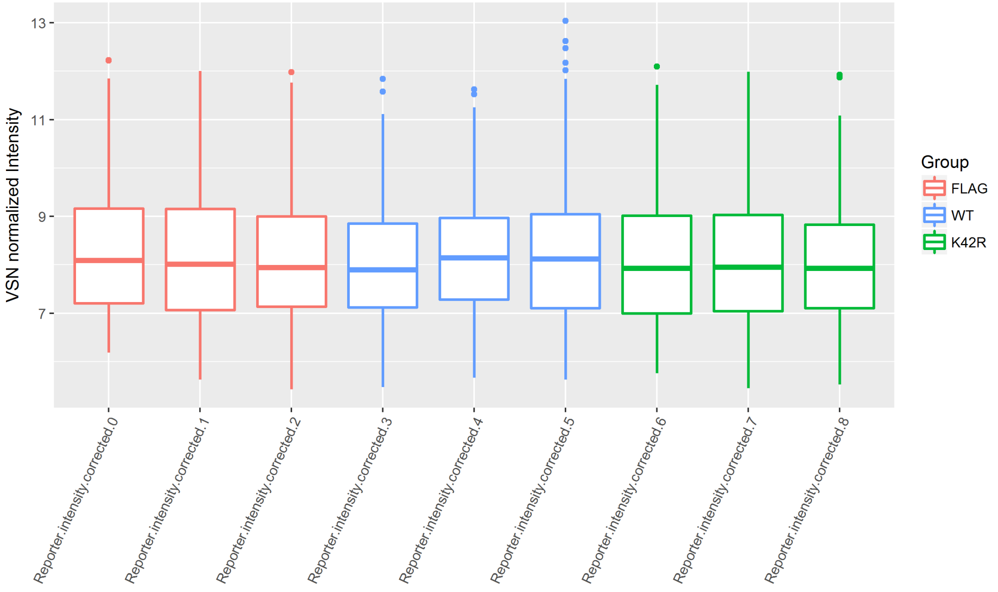

CEP131 - VSN normalized intensities over all used TMT channels

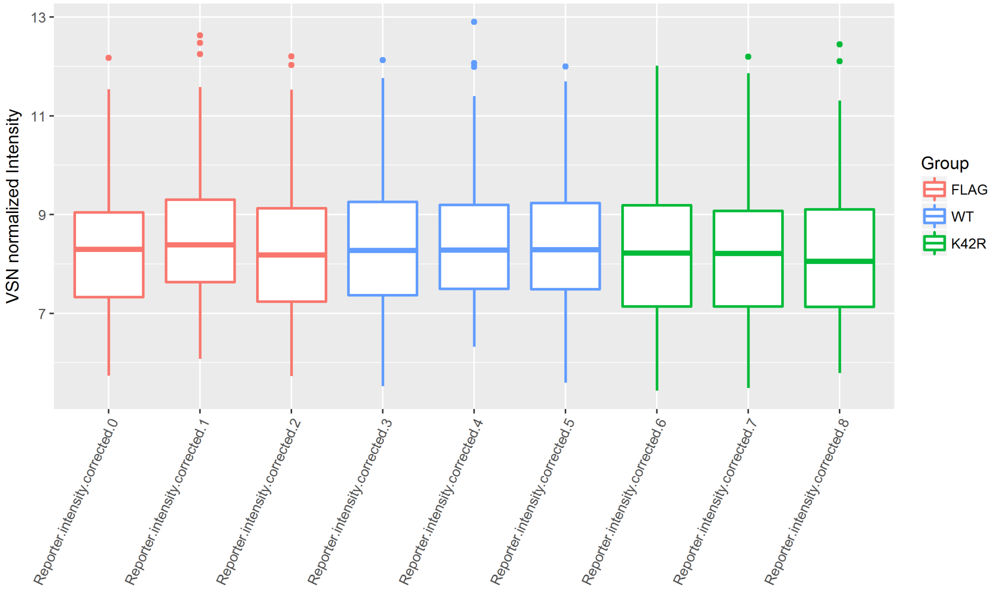

Appendix Figure S4

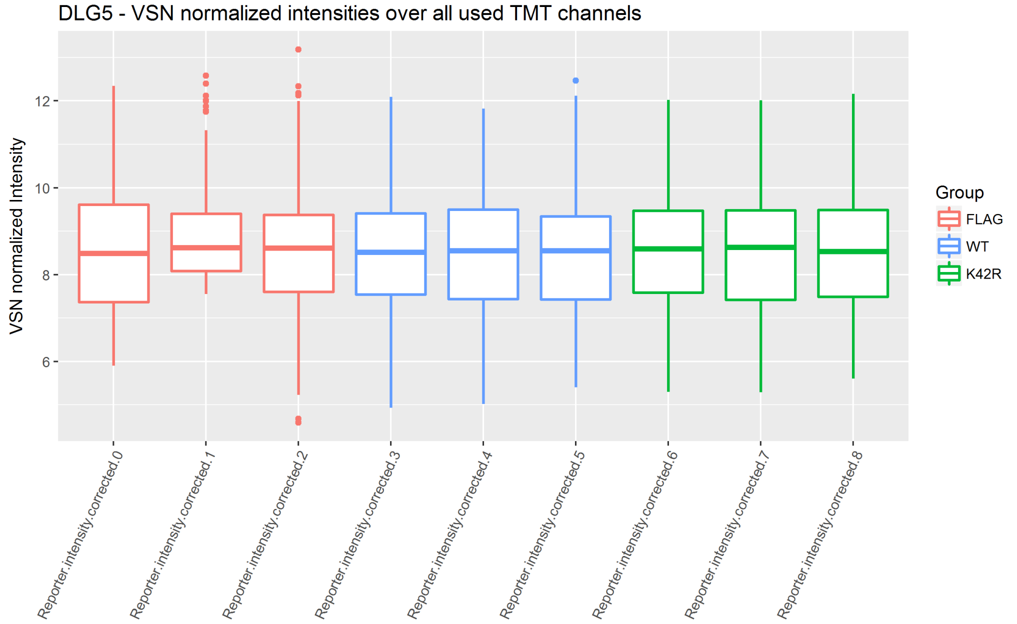

D.

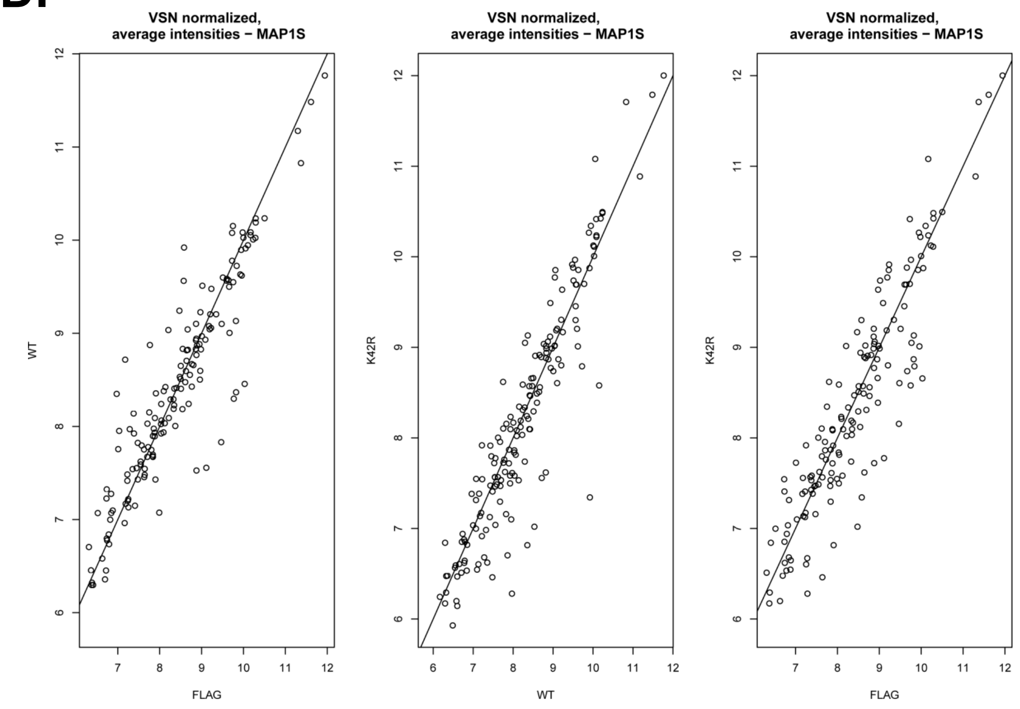

Appendix Figure S4

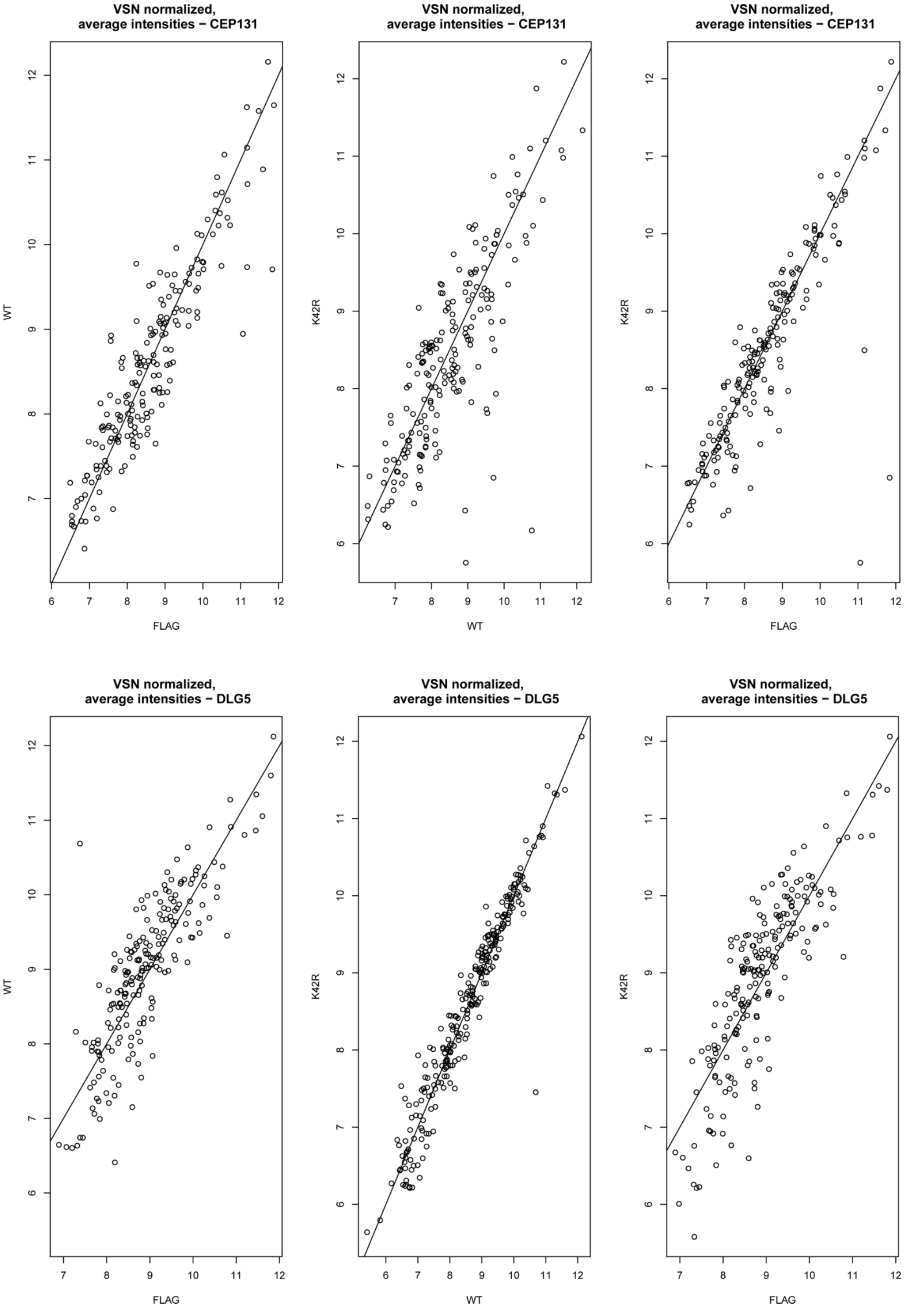

Appendix Figure S4. Data quality control of TMT labelling from FLAG-tagged MAP1S, CEP131 and DLG5 immunoprecipitates for XIC analysis

A. QQ-plots of raw data and VSN adjusted, corrected intensity TMT reporter ions for MAP1S, CEP131 and DLG5. Figures show that VSN adjusted intensities follow a normal distribution reasonably well.
B. VSN model fit for corrected TMT reporter intensities of MAP1S, CEP131 and DLG5. Figures show an approximately constant standard deviation over the ranked intensity values.
C. Boxplots of the VSN adjusted, corrected TMT reporter ion intensities of each TMT label for MAP1S, CEP131 and DLG5. No systematic intensity bias visible.
D. Scatterplots of the average VSN adjusted, corrected reporter ion intensities of identified peptides of each of the three groups (FLAG only, WT and $K^{42}R$) against each other. Intensities scatter around a 1:1 ratio (45 degree line). No obvious systematic bias of corrected TMT reporter ion intensity in the groups visible.
All graphs in Appendix Fig. S4 were computed using Appendix Script File S1.
