# Supplementary material for: Phosphoproteomic screening identifies physiological substrates of the CDKL5 kinase
Source: EMBO J. 2018 Sep 28;37(24):e99559. doi: 10.15252/embj.201899559 (PMC6293279; doi:10.15252/embj.201899559)
Supplement: Supplementary file 2 — Expanded View Figures PDF [file EMBJ-37-e99559-s002.pdf]

Expanded View Figures

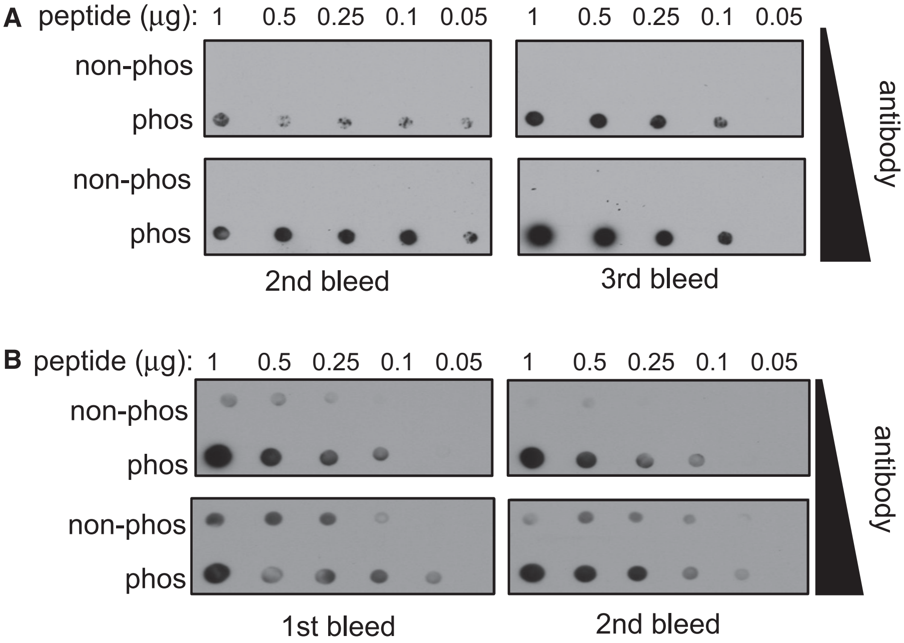

Figure EV1. Dot blot analysis of phospho-specific antibodies.

A Dot blot analysis of the MAP1S pSer^{900} antibodies. Increasing amounts of MAP1S pSer^{900} phosphopeptide immunogen and non-phosphopeptide equivalent were spotted onto nitrocellulose, and membrane was subjected to dot blotting with two different concentrations of affinity-purified MAP1S pS^{900} antibodies (0.2 µg/ml for 18 h or 1 µg/ml for 1 h). Two different antibody bleeds were tested.

B Dot blot analysis of CEP131 pSer^{35} antibodies. Increasing amounts of CEP131 pSer^{35} phosphopeptide immunogen and non-phosphopeptide equivalent was spotted onto nitrocellulose. After cross-linking with glutaraldehyde (1% (v/v) in H_2O for 10 min), the membrane was subjected to dot blotting with two different concentrations of affinity-purified CEP131 pSer^{35} antibodies (0.2 µg/ml for 18 h or 1 µg/ml for 1 h). Two different antibody bleeds were tested.

Source data are available online for this figure.

Figure EV2. CDKLs 1–4 cannot phosphorylate MAP1S or CEP131 in cells.

A Schematic representation of CDKLs 1–5. The kinase catalytic domain is highlighted in dark blue. Amino acid numbers at the N- and C-termini are indicated; the positions of conserved residues in the ATP binding sites that were mutated to render these protein “kinase-dead” are also indicated.

B HEK293 cells were co-transfected with C-terminally tagged FLAG-tagged CDKLs 1,2,3,4 or 5 (wild type “WT” or the relevant kinase-dead mutant) and HA-MAP1S. Anti-HA precipitates were subjected to Western blotting with the antibodies indicated. “Hi” higher exposure; “lo” lower exposure. The input extracts were also subjected to immunoblotting (lower panels). Three independent experiments were done, and one representative experiment is shown.

C Same as (B) except that HEK293 cells were co-transfected with C-terminally tagged FLAG-tagged CDKLs 1,2,3,4 or 5 (wild type “WT” or the relevant kinase-dead mutant) and HA-CEP131. Three independent experiments were done, and one representative experiment is shown.

D HEK293 cells were transfected with C-terminally tagged FLAG-tagged CDKLs 1,2,3,4 or 5 (wild type “WT” or the relevant kinase-dead mutant). Anti-FLAG precipitates were incubated with the MAP1S S^{900} synthetic peptide in the presence of [γ-^{32}P]-labelled ATP-Mg^{2+}, and peptide phosphorylation was measured by Cerenkov counting. Data are represented as mean ± SEM from three independent experiments.

Source data are available online for this figure.

▶

 © 2018 The Authors

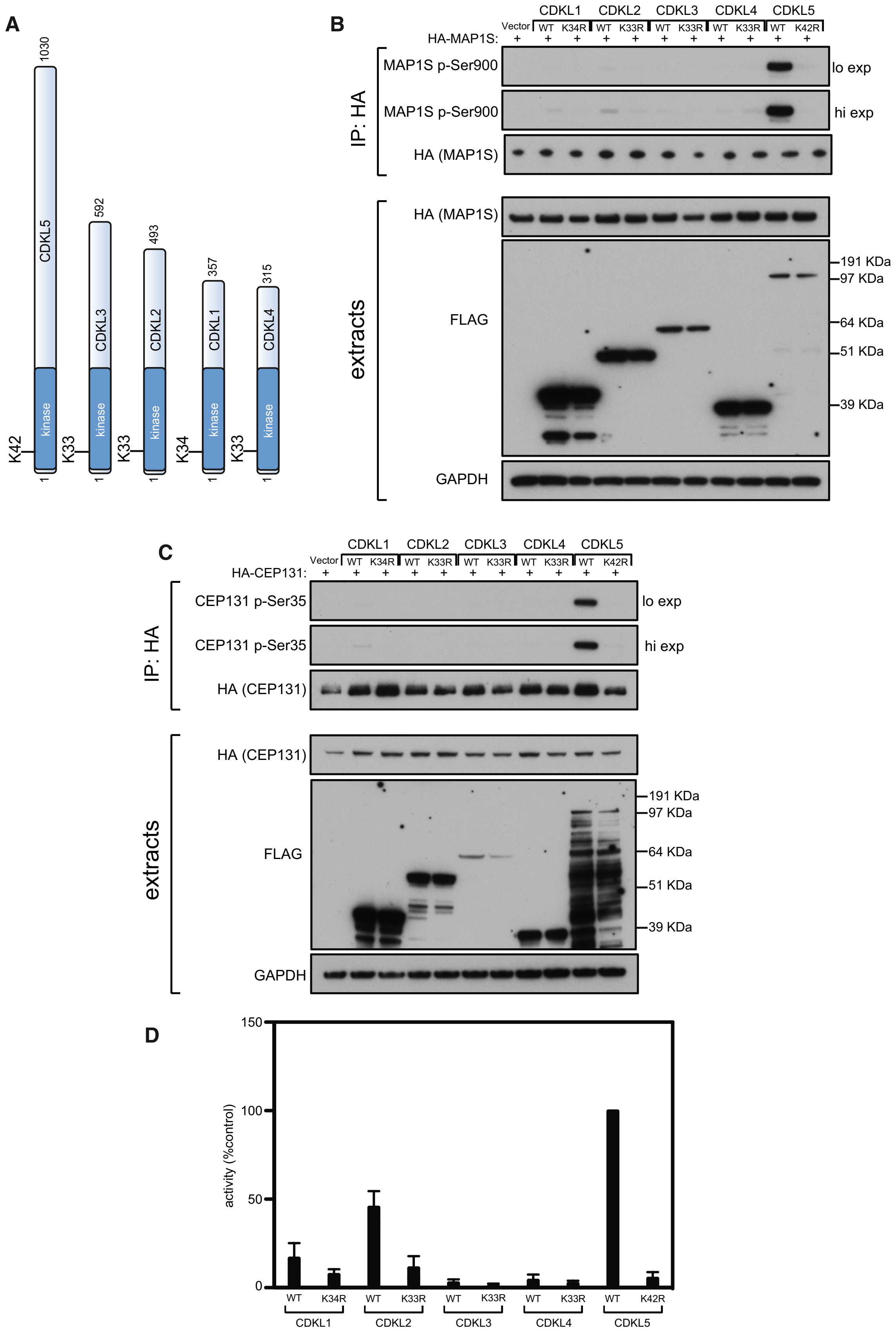
A
CDKL5
CDKL3
CDKL2
CDKL1
CDKL4
kinase
1030
592
493
357
315
K42
K33
K34
B
HA-MAP1S:
Vector
WT
K34R
K33R
K42R
IP: HA
MAP1S p-Ser900
lo exp
hi exp
HA (MAP1S)
extracts
FLAG
GAPDH
191 KDa
97 KDa
64 KDa
51 KDa
39 KDa
C
HA-CEP131:
CEP131 p-Ser35
HA (CEP131)
D
activity (%control)
150
100
50
0

Figure EV2.

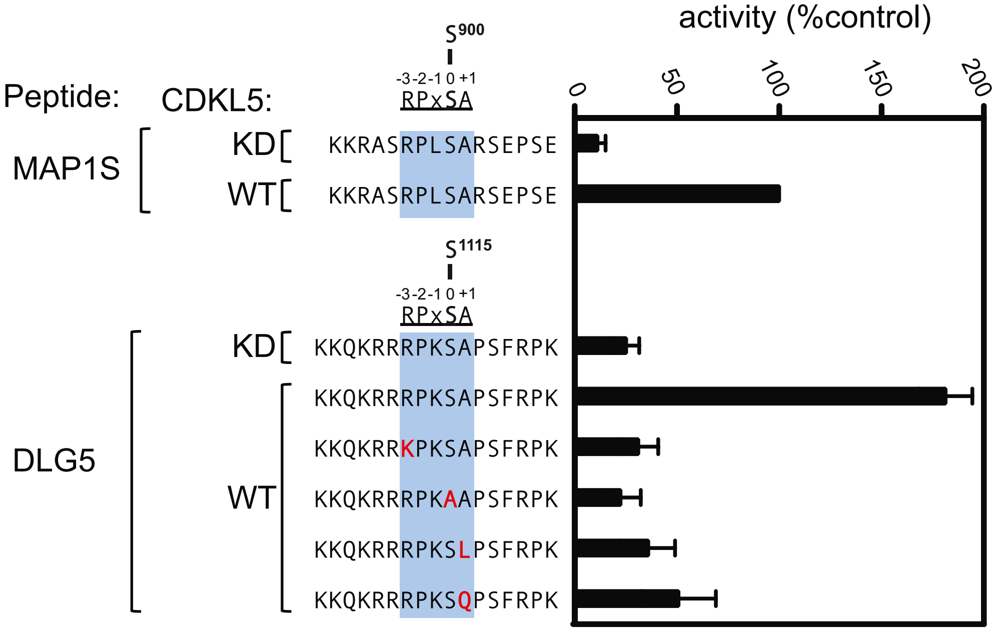

Figure EV3. CDKL5 phosphorylates DLG5 Ser^{1115} *in vitro*.

Anti-FLAG precipitates from HEK293 cells transiently expressing FLAG-tagged CDKL5 (wild type "WT" or a K^{42}R kinase-dead "KD" mutant) were incubated with the synthetic peptides indicated in the presence of [γ-^{32}P]-labelled ATP-Mg^{2+}, and peptide phosphorylation was measured by Cerenkov counting. Data are represented as mean ± SEM from three independent experiments.

Source data are available online for this figure.

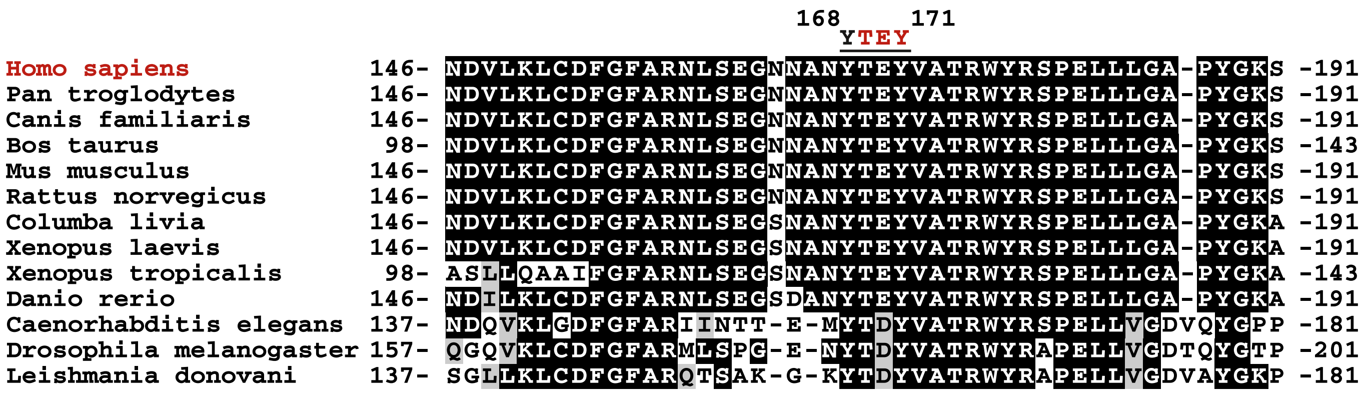

Figure EV4. Sequence conservation around the CDKL5 T-loop.

Schematic diagram showing alignment of catalytic subdomains VII and VIII from CDKL5 in species indicated. The position of the conserved ^{168}Y-TEY^{171} (human numbering) motif is indicated.

© 2018 The Authors

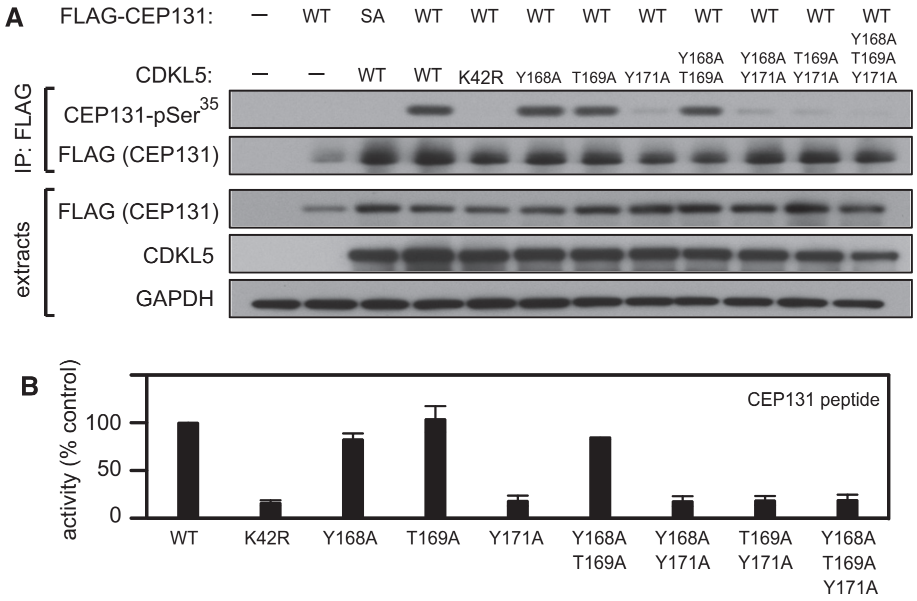

Figure EV5. T-loop autophosphorylation is critical for CDKL5 activity towards CEP131.

A HEK293 cells were co-transfected with untagged CDKL5 (wild type "WT" or the mutants indicated) and FLAG-tagged CEP131 [wild type (WT), a Ser^{35}Ala mutant (SA)] or empty vector (−). Anti-FLAG precipitates were subjected to Western blotting with the antibodies indicated. The input extracts were also subjected to immunoblotting (lower panels). Three independent experiments were done, and one representative experiment is shown.

B Anti-FLAG precipitates from HEK293 cells transiently expressing FLAG-tagged CDKL5 (wild type "WT" or the mutants indicated) were incubated with a synthetic peptide corresponding to the sequence around the CEP131 Ser^{35} phosphorylation site, in the presence of [γ-^{32}P]-labelled ATP-Mg^{2+}. Peptide phosphorylation was quantitated in a scintillation counter. Data are represented as mean ± SEM from three independent experiments.

Source data are available online for this figure.

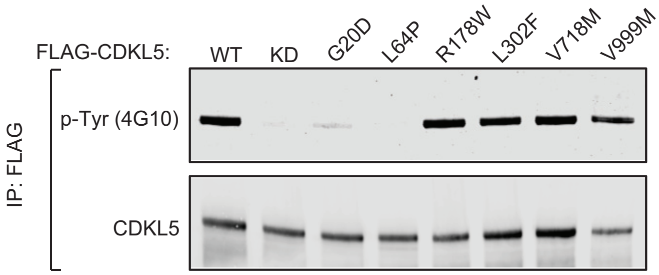

Figure EV6. Effect of pathological mutations on CDKL5 tyrosine phosphorylation.

HEK293 cells were transfected with FLAG-tagged CDKL5 (wild type "WT" or a K^{42}R kinase-dead "KD" mutant, or the mutants indicated). Anti-FLAG precipitates were subjected to Western blotting with the antibodies indicated.

Source data are available online for this figure.

© 2018 The Authors
