# Supplementary material for: Phosphoproteomic screening identifies physiological substrates of the CDKL5 kinase
Source: EMBO J. 2018 Sep 28;37(24):e99559. doi: 10.15252/embj.201899559 (PMC6293279; doi:10.15252/embj.201899559)
Supplement: Supplementary file 7 — Code EV1 [file EMBJ-37-e99559-s007.zip › Legend_R_script_EV1.rtf]

Computer Code EV1: R-script for statistical testing of XIC data  R-script used for statistical analysis and testing of MaxQuant processed MAP1S, CEP131 and DLG5 TMT mass spectrometry data. Placement of respective MaxQuant output files into their correct folders (as indicated in script) will reproduce images taken for Appendix Figure S4 and data used for GraphPad Prism to produce parts B, D and F of Figure 5.
