# Supplementary material for: Phosphoproteomic screening identifies physiological substrates of the CDKL5 kinase
Source: EMBO J. 2018 Sep 28;37(24):e99559. doi: 10.15252/embj.201899559 (PMC6293279; doi:10.15252/embj.201899559)

Figure EV1

A.

MAP1S 2nd bleed high Ab

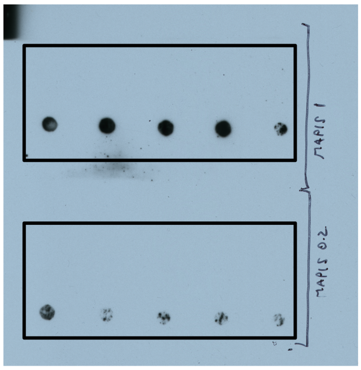

MAP1S 2nd bleed low Ab

MAP1S 3rd bleed high Ab

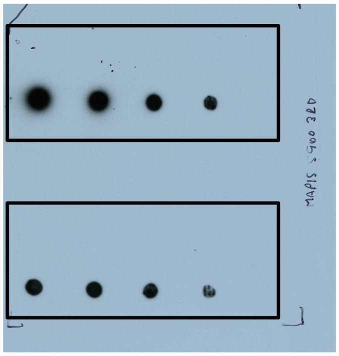

MAP1S 3rd bleed low Ab

B.

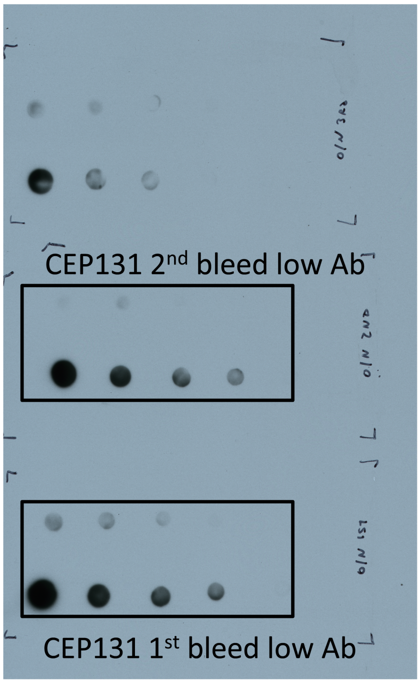

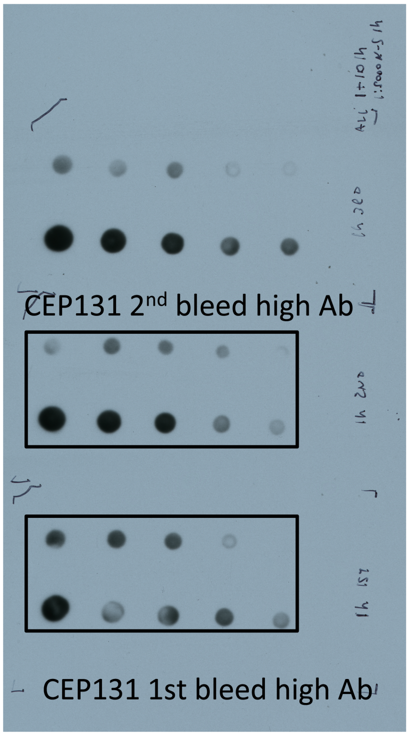

Supplement: Supplementary file 8 — Source Data for Expanded View [file EMBJ-37-e99559-s014.zip › Source_data_Fig_EV1.pdf]
