# Supplementary figures and images for: Phosphoproteomic screening identifies physiological substrates of the CDKL5 kinase
Source: EMBO J. 2018 Sep 28;37(24):e99559. doi: 10.15252/embj.201899559 (PMC6293279; doi:10.15252/embj.201899559)

Figure EV5

A.

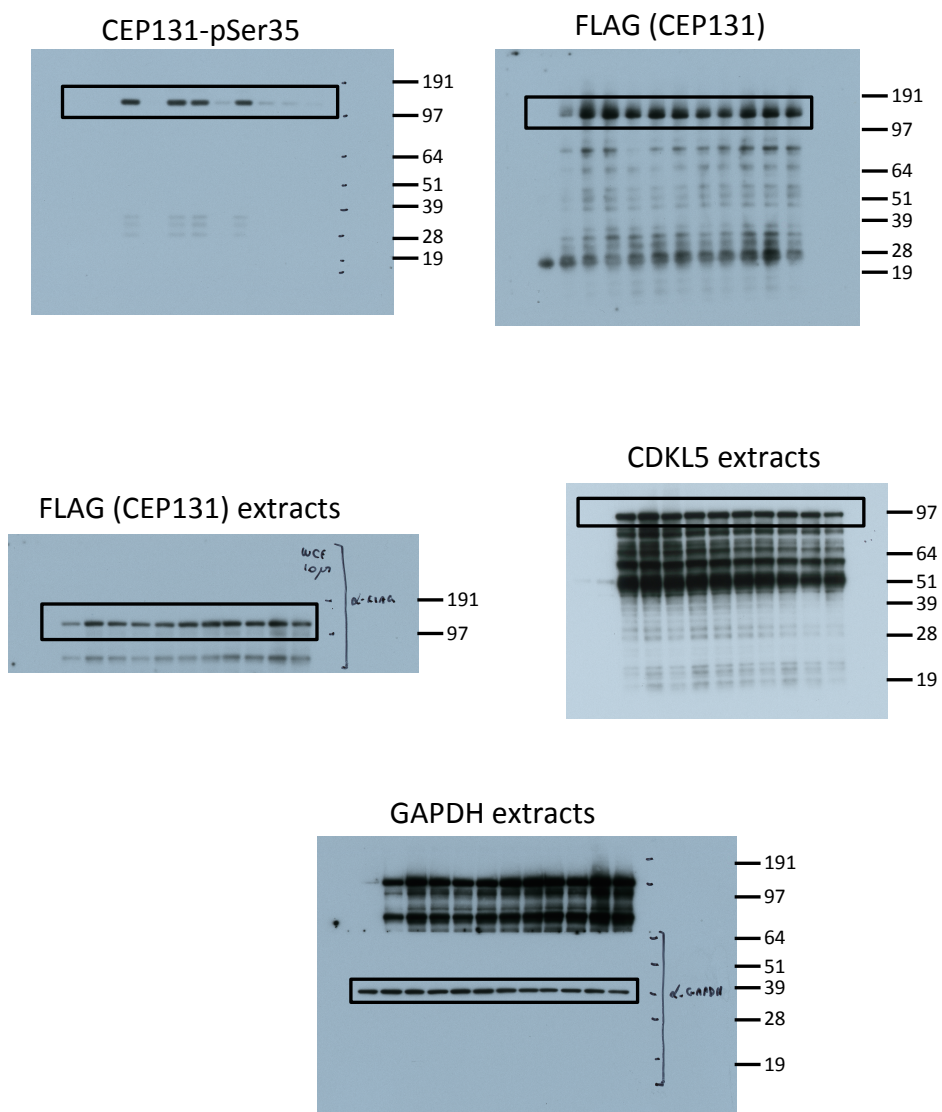

Supplement: Supplementary file 8 — Source Data for Expanded View [file EMBJ-37-e99559-s014.zip › Source_data_Fig_EV5.pdf]

**B.**

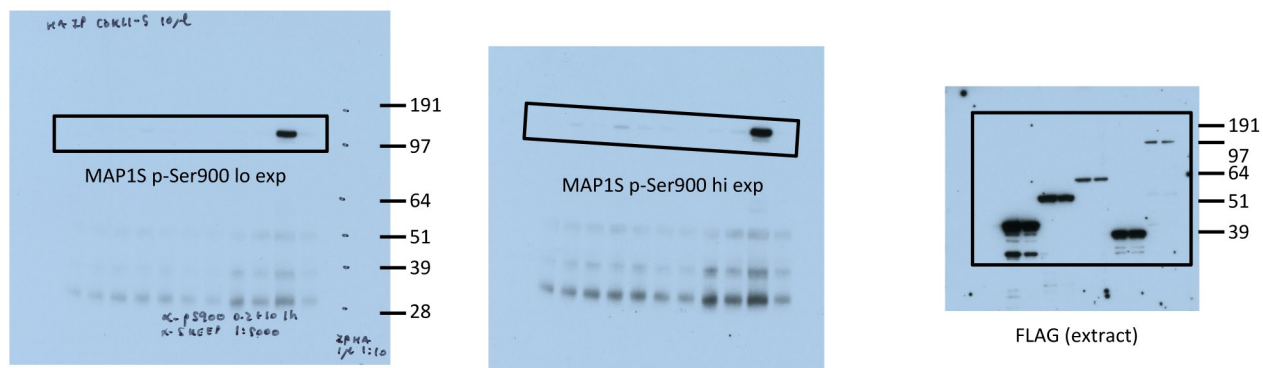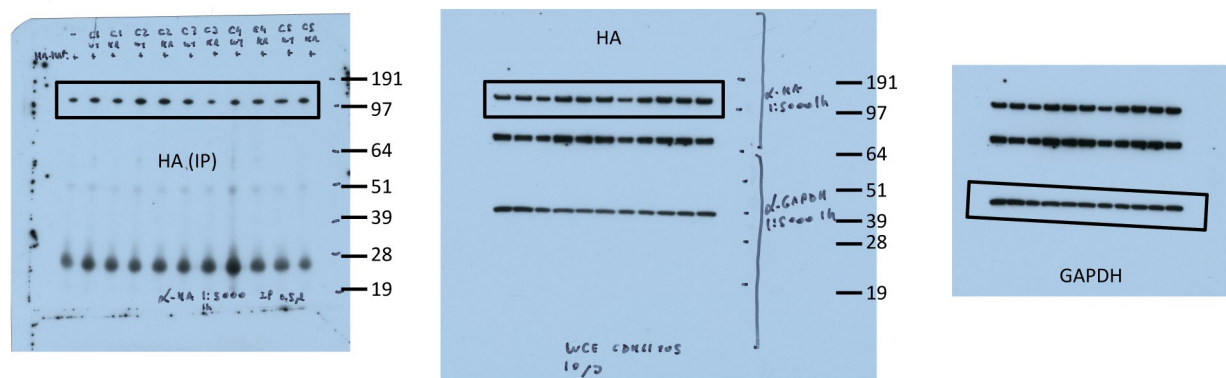

C.

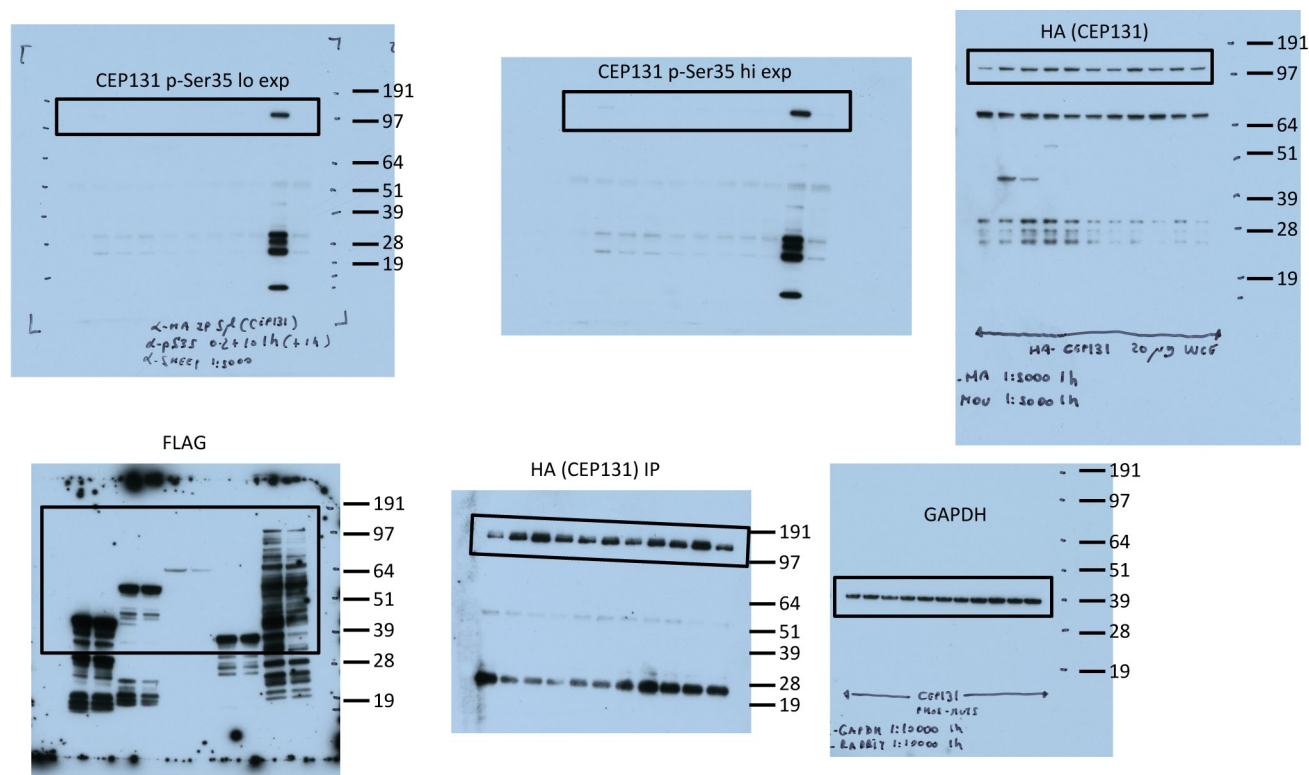

Supplement: Supplementary file 8 — Source Data for Expanded View [file EMBJ-37-e99559-s014.zip › Source_data_Fig_EV2.pdf]

Figure EV6

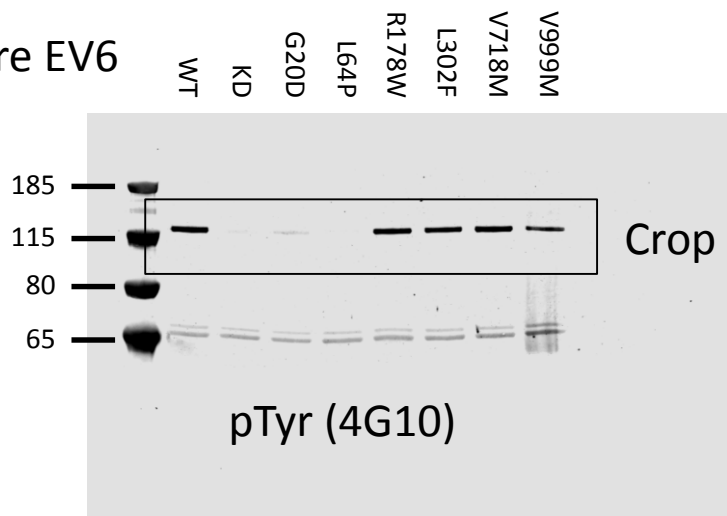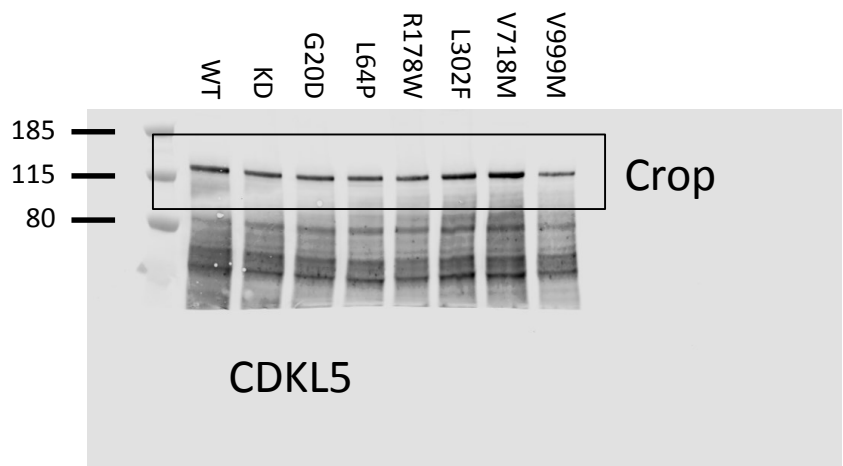

Supplement: Supplementary file 8 — Source Data for Expanded View [file EMBJ-37-e99559-s014.zip › Source_data_Fig_EV6.pdf]

Figure 1

B.

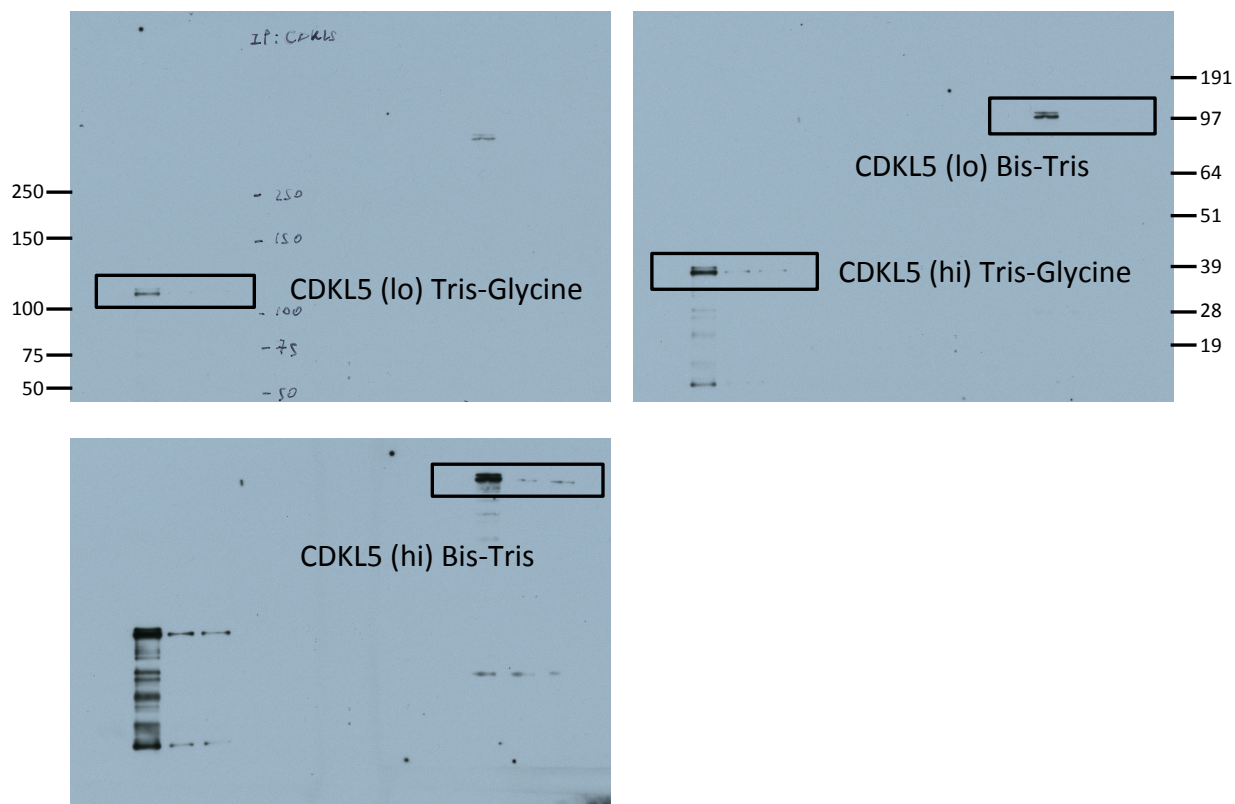

C.

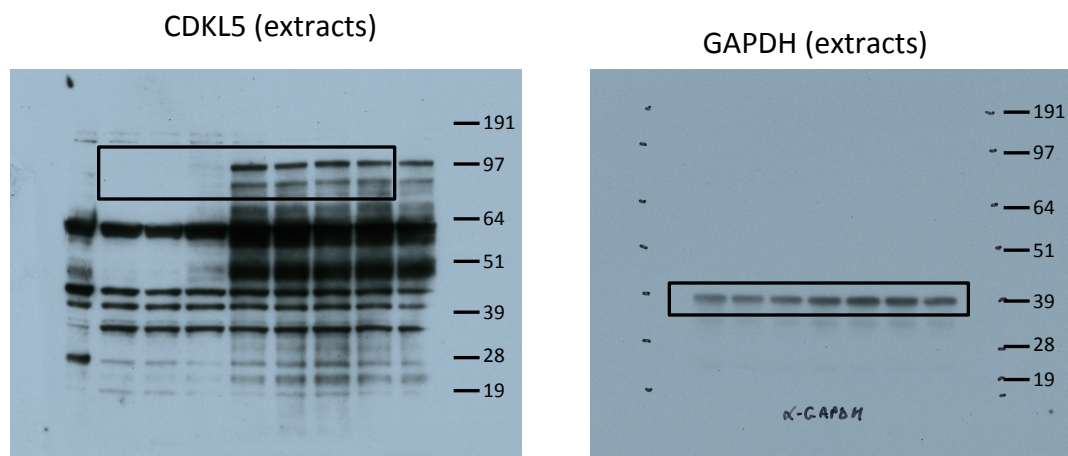

Supplement: Supplementary file 10 — Source Data for Figure 1 [file EMBJ-37-e99559-s008.pdf]

Figure 3

A.

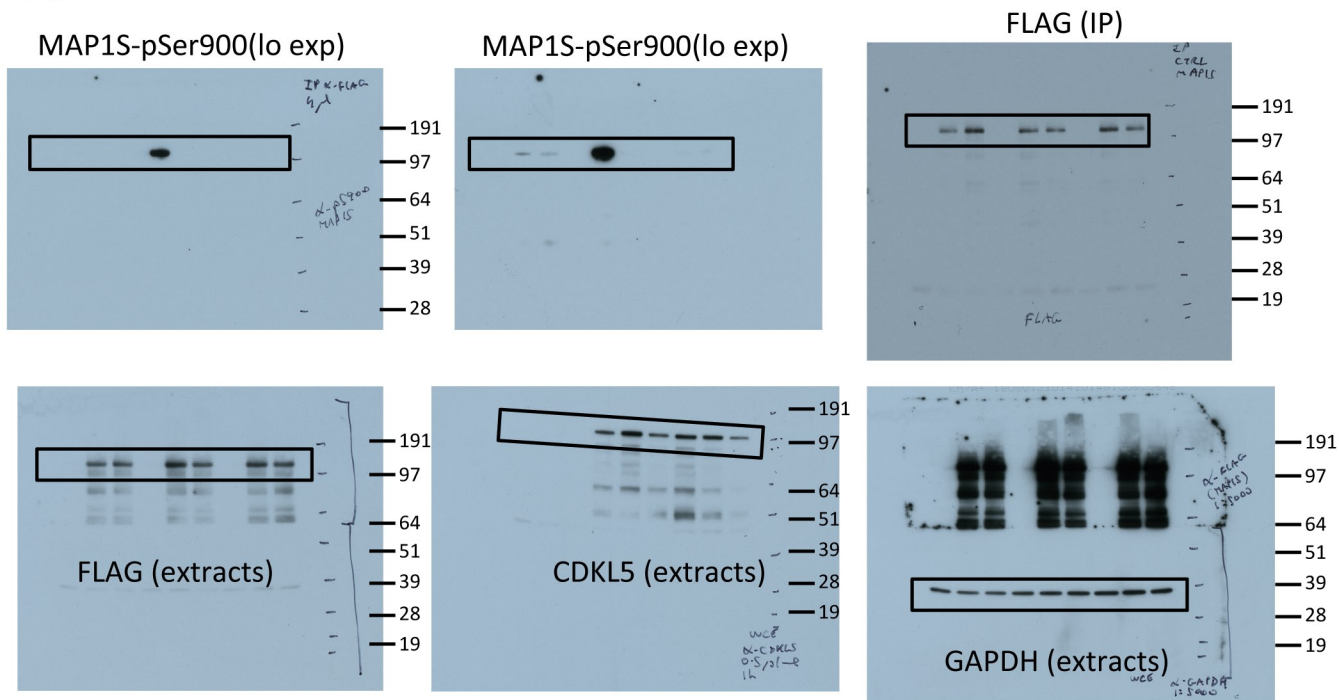

B.

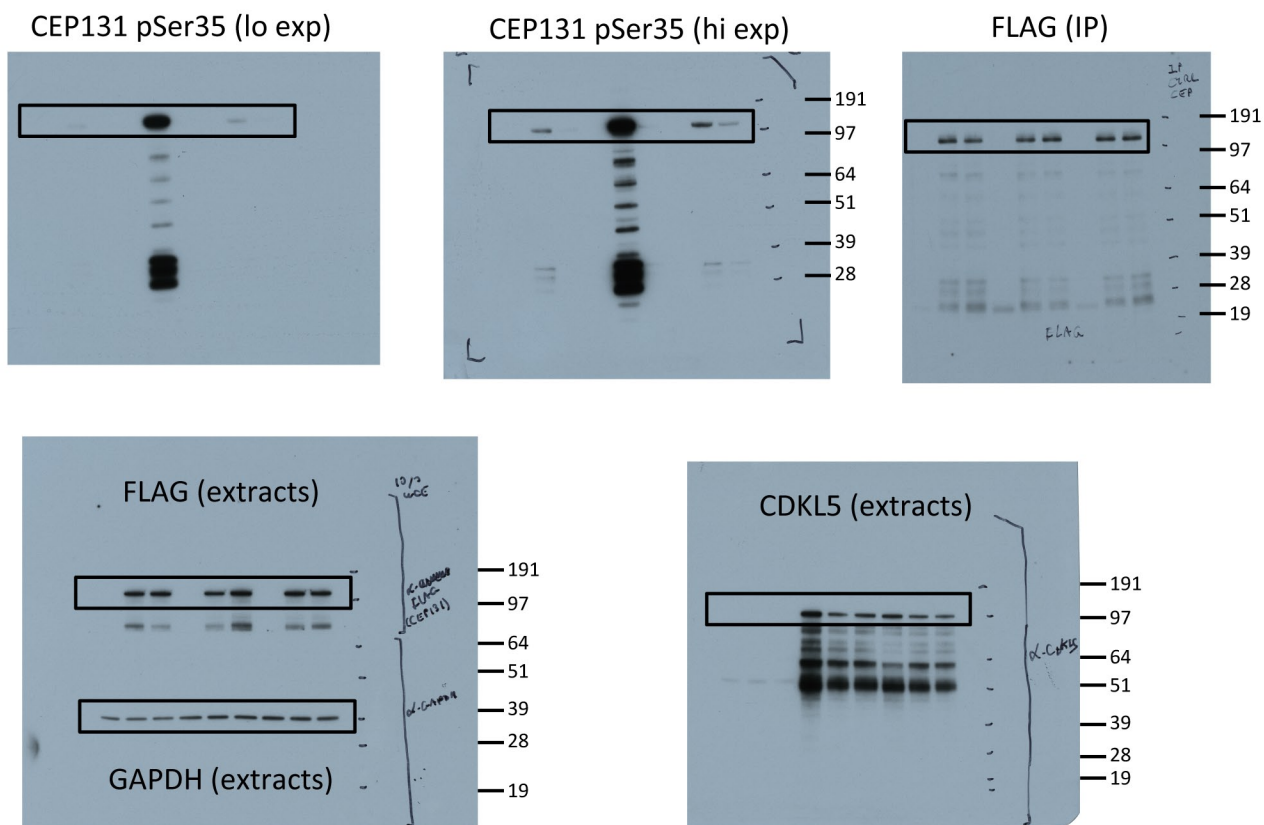

Supplement: Supplementary file 11 — Source Data for Figure 3 [file EMBJ-37-e99559-s009.pdf]

Figure 5

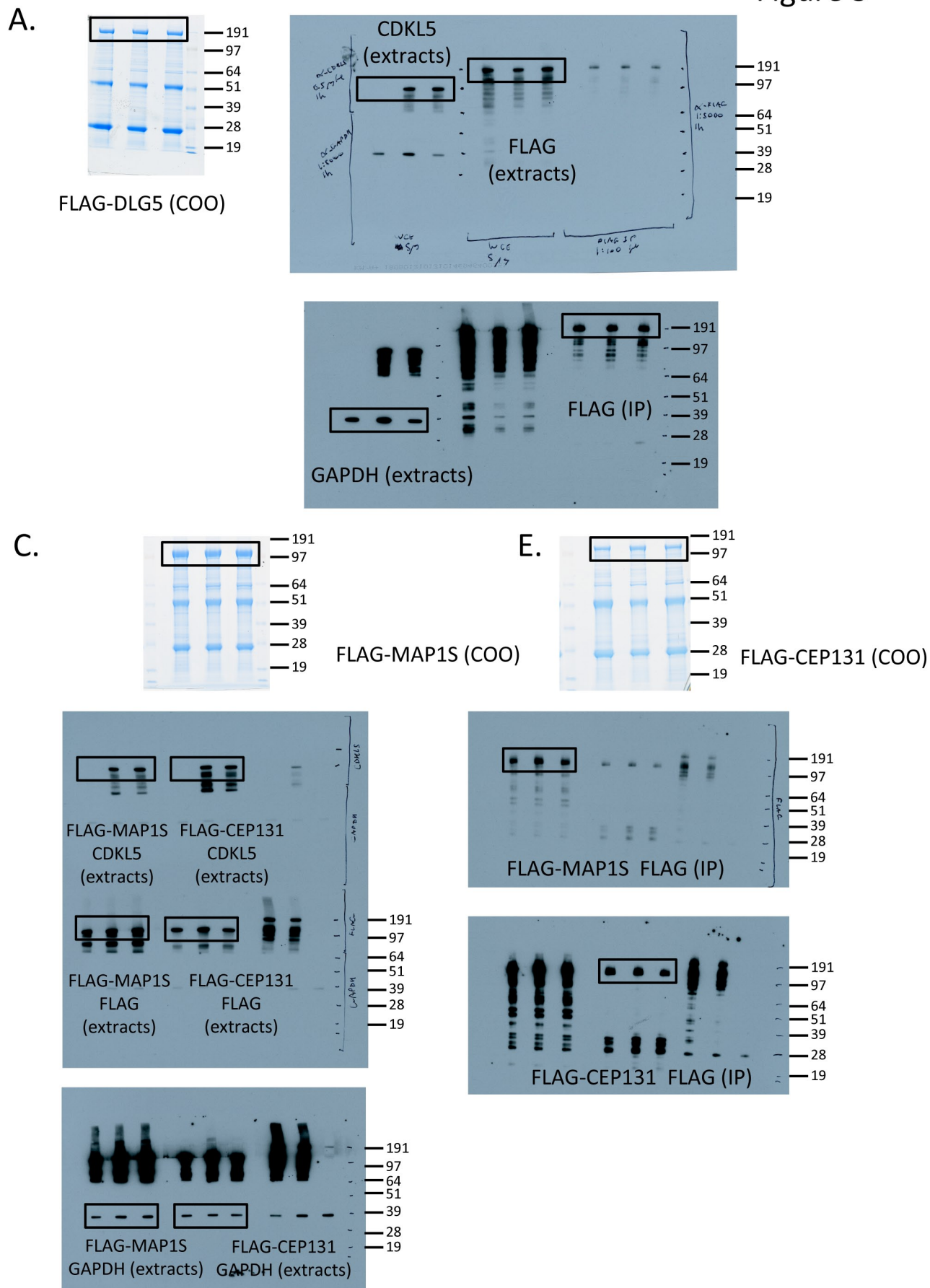

Supplement: Supplementary file 13 — Source Data for Figure 5 [file EMBJ-37-e99559-s011.pdf]

Figure 6

A.

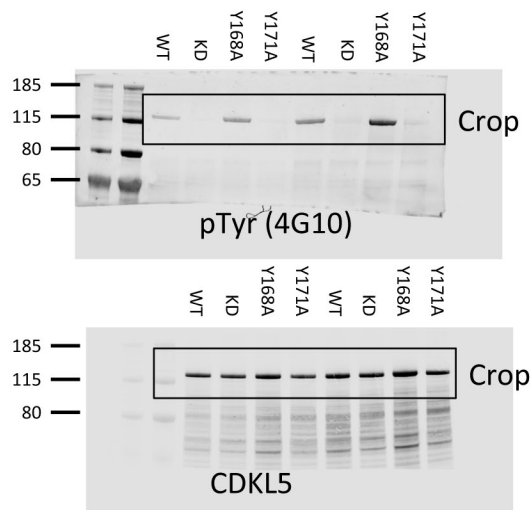

B.

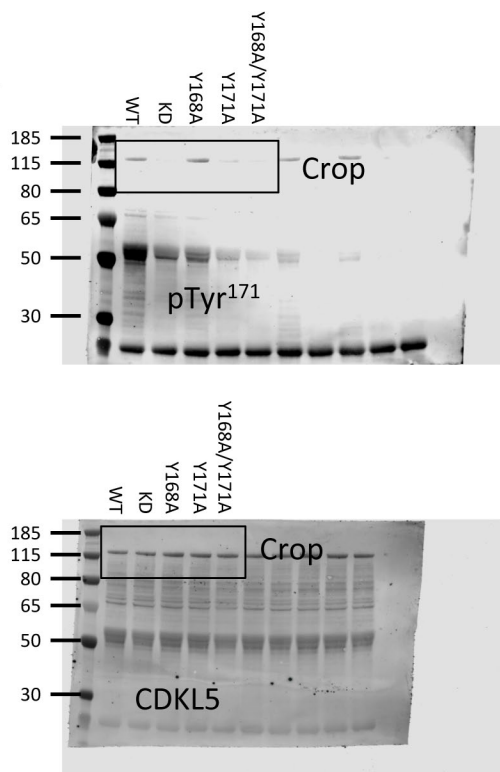

E.

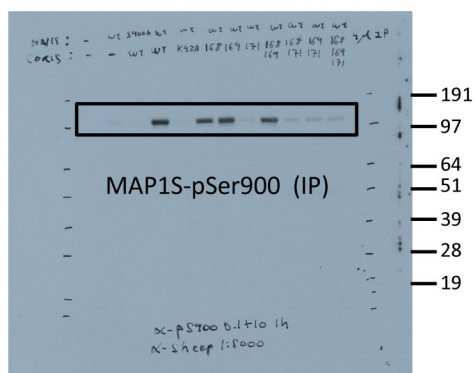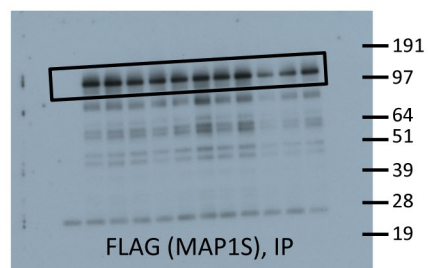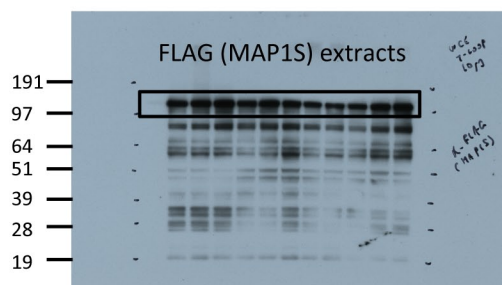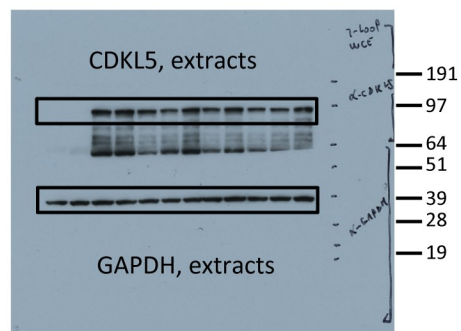

Supplement: Supplementary file 14 — Source Data for Figure 6 [file EMBJ-37-e99559-s012.zip › Source_data_Fig6.pdf]

Figure 7

A.

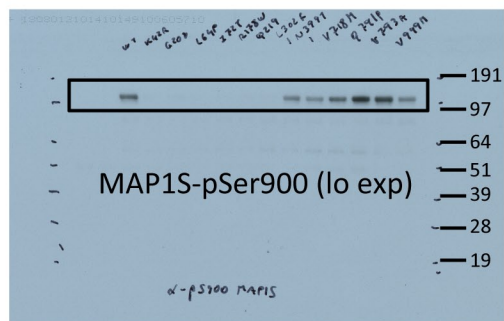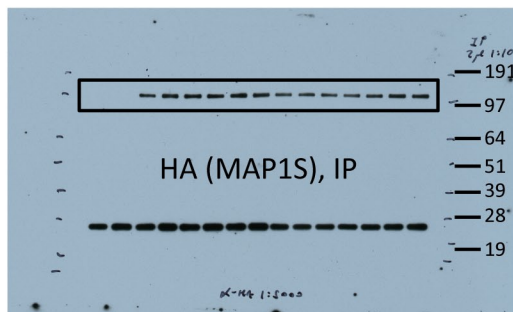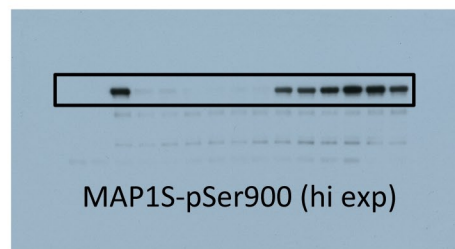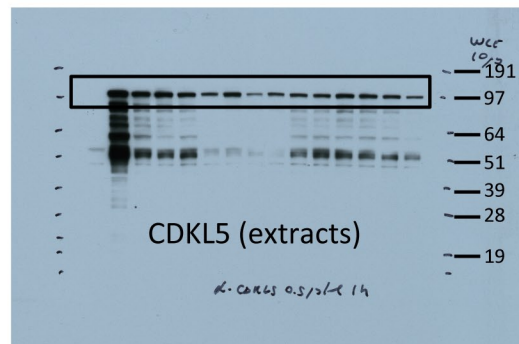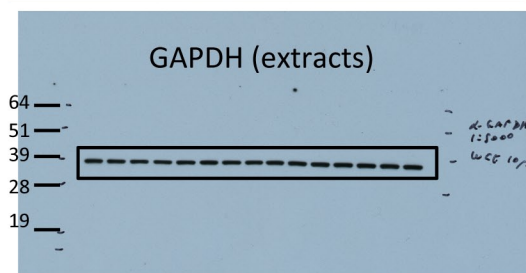

C.

CEP131-pSer35 (lo exp)

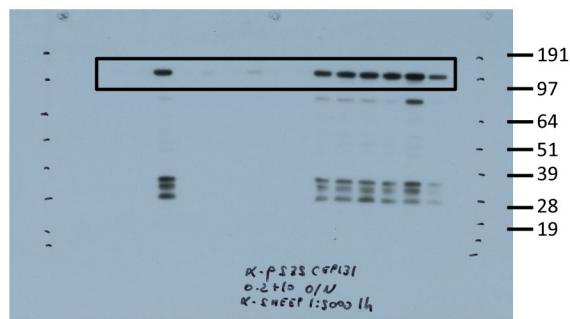

CEP131-pSer35 (hi exp)

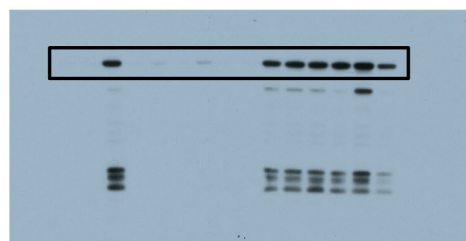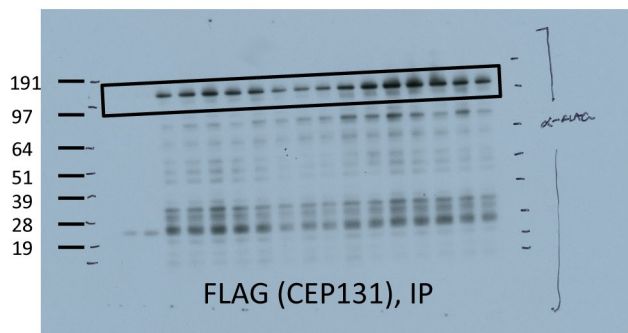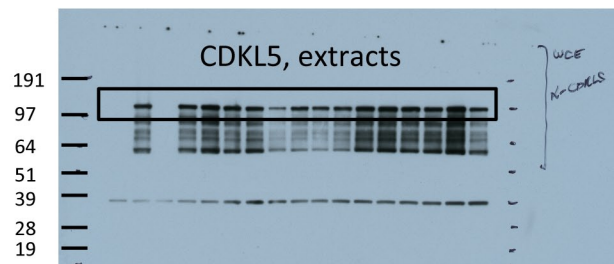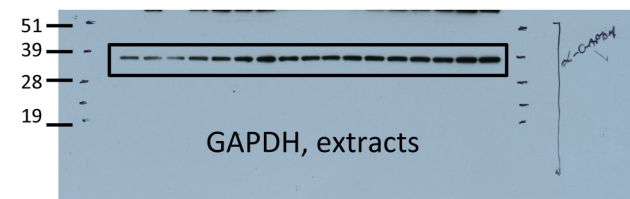

Supplement: Supplementary file 15 — Source Data for Figure 7 [file EMBJ-37-e99559-s013.zip › Source_data_Fig7.pdf]
